# T2-mapping increase is the prevalent imaging biomarker of myocardial involvement in active COVID-19: a Cardiovascular Magnetic Resonance study

**DOI:** 10.1186/s12968-021-00764-x

**Published:** 2021-06-10

**Authors:** Nicola Galea, Livia Marchitelli, Giacomo Pambianchi, Federica Catapano, Giulia Cundari, Lucia Ilaria Birtolo, Viviana Maestrini, Massimo Mancone, Francesco Fedele, Carlo Catalano, Marco Francone

**Affiliations:** 1 Department of Experimental Medicine, “Sapienza” University of Rome, Viale del Policlinico 155, 00161 Rome, Italy; 2 Department of Radiological, Oncological and Pathological Sciences, “Sapienza” University of Rome, Viale del Policlinico 155, 00161 Rome, Italy; 3 Department of Cardiovascular and Respiratory Diseases, “Sapienza” University of Rome, Viale del Policlinico 155, 00161 Rome, Italy; 4 Department of Biomedical Sciences, Humanitas University, Via Rita Levi Montalcini 4, 20072 Pieve Emanuele, MI Italy

**Keywords:** COVID-19, SARS-CoV-2, Troponin, Cardiovascular Magnetic Resonance, Myocarditis, Inflammation

## Abstract

**Background:**

Early detection of myocardial involvement can be relevant in coronavirus disease 2019 (COVID-19) patients to timely target symptomatic treatment and decrease the occurrence of the cardiac sequelae of the infection.

The aim of the present study was to assess the clinical value of cardiovascular magnetic resonance (CMR) in characterizing myocardial damage in active COVID-19 patients, through the correlation between qualitative and quantitative imaging biomarkers with clinical and laboratory evidence of myocardial injury.

**Methods:**

In this retrospective observational cohort study, we enrolled 27 patients with diagnosis of active COVID-19 and suspected cardiac involvement, referred to our institution for CMR between March 2020 and January 2021.

Clinical and laboratory characteristics, including high sensitivity troponin T (hs-cTnT), and CMR imaging data were obtained. Relationships between CMR parameters, clinical and laboratory findings were explored.

Comparisons were made with age-, sex- and risk factor–matched control group of 27 individuals, including healthy controls and patients without other signs or history of myocardial disease, who underwent CMR examination between January 2020 and January 2021.

**Results:**

The median (IQR) time interval between COVID-19 diagnosis and CMR examination was 20 (13.5–31.5) days. Hs-cTnT values were collected within 24 h prior to CMR and resulted abnormally increased in 18 patients (66.6%). A total of 20 cases (74%) presented tissue signal abnormalities, including increased myocardial native T1 (n = 11), myocardial T2 (n = 14) and extracellular volume fraction (ECV) (n = 10), late gadolinium enhancement (LGE) (n = 12) or pericardial enhancement (n = 2). A CMR diagnosis of myocarditis was established in 9 (33.3%), pericarditis in 2 (7.4%) and myocardial infarction with non-obstructive coronary arteries in 3 (11.11%) patients. T2 mapping values showed a moderate positive linear correlation with Hs-cTnT (r = 0.58; p = 0.002). A high degree positive linear correlation between ECV and Hs-cTnT was also found (r 0.77; p < 0.001).

**Conclusions:**

CMR allows in vivo recognition and characterization of myocardial damage in a cohort of selected COVID-19 individuals by means of a multiparametric scanning protocol including conventional imaging and T1–T2 mapping sequences. Abnormal T2 mapping was the most commonly abnormality observed in our cohort and positively correlated with hs-cTnT values, reflecting the predominant edematous changes characterizing the active phase of disease.

**Supplementary Information:**

The online version contains supplementary material available at 10.1186/s12968-021-00764-x.

## Background

Myocardial injury is not uncommon in coronavirus disease 19 (COVID-19) and recognizes a complex multifactorial pathogenesis, including direct viral toxicity, uncontrolled immune-activation (the so called “cytokine storm”), stress cardiomyopathy, mismatch ischemia and prothrombotic activation with plaques formation and microvascular disease [1, 2]. By definition, the term refers to any patient presenting with at least one cardiac troponin (cTnT) concentration above the 99th percentile upper reference limit [3]. Reported rates of increased cTnT levels range from 7 to 36% of COVID-19 inpatients and are associated with higher prevalence of cardiovascular diseases (CVD) [1, 4, 5].

This heterogeneity probably relies on the different thresholds applied in cTnT assays as well as on the clinical severity of the cases included, which has shown to be significantly related with the extent of myocardial damage. A recent metanalysis summarized that acute myocardial injury rate was 13-fold higher in intensive care unit patients as compared to those with mild forms of infection [6]. Nevertheless, limited evidence exists on the assessment of myocardial damage in patients with mild disease.

Whilst it remains debated whether cTnT rise necessarily reflects direct cardiac infection, there is a clear independent association between myocardial injury and mortality rate. Fatal outcomes were reported in 37.5% in patients with elevated levels of cTnT and raised to nearly 70% in the presence of pre-existing CVD comorbidities [7]. These data were confirmed by two independent studies reporting sudden cardiac arrest as fatal outcomes in both in-hospital [8] and out-hospital [9] settings/frameworks.

Despite the evidence, the American College of Cardiology does not recommend routine testing of cTnT levels in COVID-19 patients, unless the diagnosis of acute myocardial infarction (MI) is suspected on clinical grounds [10].

This potentially excludes from the screening most of pauci- or asymptomatic patients in which myocardial injury yields prognostic significance.

In this scenario, early detection of myocardial involvement can be relevant to timely target symptomatic treatment and decrease the occurrence of the devastating cardiac sequelae of the infection.

Our endpoint is to assess the clinical value of a non-invasive and highly sensitive tool like cardiovascular magnetic resonance (CMR) in characterizing myocardial damage in COVID-19 patients, through the correlation of qualitative and quantitative CMR features with clinical and laboratory evidence of myocardial injury. Our quantitative analysis relies on the use of parametric mapping, which is an innovative and reproducible method to provide unique quantitative data about T1 and T2 relaxation time changes in the myocardium.

As a further advantage, CMR may by integrated by the comprehensive assessment of heart, pulmonary vessels and lung parenchyma in a one-stop-shop approach, which makes it potentially suitable to contemporary exclude thromboembolic complications and follow-up pulmonary disease progression in COVID-19 patients, using a radiation-fee imaging procedure [11].

## Methods

### Study population

This was a single-center observational retrospective study on a cohort of 27 patients with a confirmed active COVID-19 by reverse transcription–polymerase chain reaction (RT-PCR) on nasopharyngeal swabs.

Patients were considered eligible if RT-PCR nasopharyngeal swab, collected within 72 h prior to CMR scan, confirmed severe acute respiratory syndrome coronavirus 2 (SARS-CoV-2) infection, COVID-19 related clinical signs and symptoms were persistent and at least one the following inclusion criteria was fulfilled:

- at least one high sensitive Troponin T (hs-cTnT) measurement above 99th percentile (> 0.014 ng/ml) in absence of ST-elevation or other signs of MI;
- newly observed reduced (< 50%) left ventricle (LV) ejection fraction (LVEF) detected by a point of care bedside echocardiography;
- no obstructive coronary artery disease (CAD) at coronary angiography despite infarct-like clinical presentation.

Exclusion criteria were general contraindications to CMR, unstable clinical conditions or inability to perform repeated breath-holds.

In all patients, the following routine blood tests and arterial blood gas tests were collected in the 48 h before the CMR examination: C-reactive protein (CRP), D-dimer, white blood cells (WBC) count, lymphocyte count, and arterial oxygen partial pressure/fraction of inspired oxygen (PaO_2/_FiO_2_) ratio.

In addition, all participants’ blood samples collected 24 h prior to CMR were processed using standardized commercially available test kits for analysis of hs-cTnT and values above 99th percentile (0.014 ng/ml) were considered abnormal. Data on respiratory rate and oxygen saturation were also reported.

Disease severity score was evaluated for all individuals, using the Chinese Center of Disease Control (CDC) criteria [12]: mild, severe and critical categories were assigned accordingly. Besides, according to the timing of symptoms’ onset, patients were classified to have an early (0–7 days) or late stage disease (> 7 days) [13]. Furthermore, the time interval between SARS-CoV-2 infection diagnosis and the CMR exam date was calculated.

Based on computed tomography (CT) scoring by Pan et al. [14], a semi-quantitative evaluation of pulmonary involvement was performed and a global CT score (0–25) was obtained by adding a single score (0–5) per each pulmonary lobe [15].

As explained elsewhere, chest CT scans were performed on two multidetector scanners (Somatom Sensation 16 and Somatom Sensation 64; Siemens Healthineers, Erlangen, Germany). Acquisition parameters were settled according to manufacturers’ recommendations for standard chest CT examination and images reconstruction was performed with filtered back-projection at 1.00 mm thickness, with a B20 kernel for soft tissue and a B60 kernel for pulmonary parenchyma [15].

A group of 27 individuals, including healthy controls and patients without clinical signs or history of myocardial disease, who underwent a CMR between January 2020 and January 2021, was also enrolled. All patients were matched for age, sex and cardiovascular risk factors (hypertension, diabetes, smoking, dyslipidemia) and performed the same CMR protocol on the same scanner as for COVID patients.

### Access to CMR scanner and sanitation procedures

All CMR examinations were performed between March 2020 and January 2021 on a fully dedicated COVID-19 1.5 T CMR scanner (Avanto, Siemens Healthineers) equipped with SQ-engine gradients (amplitude: 45 mT/m; slew rate: 200 mT/m/ms) and a 16-channel phased-array cardiac coil. According to international recommendations [16], staff members were strictly limited to three individuals, one technologist and one radiologist in the control room and one nurse in the scanning room.

A full set of personal protective equipment (PPE), including FFP2 mask, gloves, gown, goggles and/or face shield was provided to all exposed health care professionals, who were trained on the correct use of PPE.

Sanitation of the CMR facility was performed at the end of the dedicated CMR session. In case of known or suspected bacterial superinfection, cleaning between scanning two SARS-CoV-2 positive patients was carried out.

### CMR scanning protocol

All patients enrolled gave written informed consent to participate in the study.

A dose of 0.15 mmol/kg of contrast media (gadoteric acid, Claricyclic, General Electric Healthcare, Waukesha, Wisconsin, USA) was injected intravenously at a flow rate of 2.5 ml/s.

According to Society for Cardiovascular Magnetic Resonance recommendation [17] CMR protocol included:

-Black blood T2-weighted short Tau Inversion-recovery (STIR) images acquired on multiple cardiac axes, including a stack of short axis views covering the entire LV.
-Modified Look-Locker inversion-recovery (MOLLI) images acquired before and 15 min after gadolinium injection on three matched short-axis slices at basal, mid and apical views and one four-chamber view.
-T2-prepared True-FISP (T2 map) images acquired on three matched short-axis slices at basal, mid and apical planes and one four-chamber view.
-Balanced steady state free precession cine-CMR (bSSFP cine-CMR) images acquired after gadolinium administration in short-axis (a stack of contiguous planes from the base to the apex), 2-chamber, 4-chamber and 3-chamber planes.
-Contrast-enhanced inversion recovery T1-weighted (IR-CE T1w) images acquired from 15 to 20 min after gadolinium injection, during breath-hold at end-diastole.

CMR sequences parameters are reported in supplementary material (Additional file 1: Table S1).

In a subgroup of four patients who has proven to be poorly cooperative during the examination, contrast-enhanced MOLLI sequences were waived and IR-CE-T1 weighted (T1w) sequences were anticipated to ensure the proper acquisition of late gadolinium enhancement (LGE) images.

In selected patients, when clinically indicated, we embedded chest sequences in the CMR protocol, in order to perform a comprehensive cardio-thoracic CMR evaluation, as described elsewhere [11].

### Images analysis

CMR images were analyzed in consensus by two experienced cardiovascular radiologists with 3 and 10 years of experience, using a commercially available workstation (cvi^42^© (version 5.3.0, Circle Cardiovascular Imaging Inc., Calgary, Alberta, Canada), blinded to the subject status (COVID patient vs control).

LV volumes and mass were calculated from the short-axis bSSFP cine images.

Extracellular volume fraction (ECV) maps were generated by combining MOLLI images acquired before and 15 min after gadolinium administration as demonstrated elsewhere [18].

Myocardial native T1, T2 and ECV values were assessed by manually tracing subepicardial and subendocardial contours throughout the whole myocardial circumference on short axis images, carefully excluding the epicardial fat and ventricular cavity, on respective maps per each slice. The highest native T1, T2 and ECV values among all slices were considered for each patient.

LGE was identified with a signal intensity (SI) > 5 standard deviation (SD) as compared to the remote myocardium [19]. According to LGE distribution pattern, myocardial fibrosis/necrosis was classified as “ischemic” (subendocardial or transmural extension) or “non-ischemic” (subepicardial or mid-myocardial) [20]. Myocardial edema was identified on STIR images as areas of SI increase 2 SD above the remote myocardium or with a myocardium-to-skeletal muscle T2 ratio ≥ 1.9 [21].

Abnormal native T1, T2 and ECV parameters were defined by having a value beyond a predefined threshold (T1 > 1027 ms, T2 > 49.9 ms, and ECV > 29.5%), which correspond to the 95th percentile values of a large age- and gender- matched healthy control group retrospective recruited from our database, already examined in our center and previously selected to assess center-specific normal range.

According to new Lake Louis criteria [22], myocarditis diagnosis was established whether at least one T2-based and one T1-based criteria were present.

### Statistical analysis

Data are presented as counts and percentages for categorical data and means or medians for continuous data. Normal distribution of all variables was tested using Kolmogorov–Smirnov and the Shapiro–Wilk tests.

Pearson correlation coefficient was used to analyse the linear correlation between continuous variables. T-test for independent samples was applied to evaluate the relationship between a continuous and a categorical variable and to compare the means of two groups.

Comparisons between the groups were performed using Mann–Whitney U test for non-normal distributions.

All the quantitative parameters (hs-cTnT, native T1, T2, ECV, etc.) were studied as categorical variables by dichotomizing them in “altered” and “normal” in order to investigate the relationship between abnormal clinical and imaging features.

Chi-squared (X^2^) test was performed for the assessment of dependency between two categorical variables.

To evaluate the correspondence between hs-cTnT and T2 values, a linear regression model was carried out. A receiver operator characteristic (ROC) curve was used to determine the diagnostic accuracy for hs-cTnT in predicting myocardial involvement assessed by T2 mapping.

Youden’s test was applied to identify the optimal hs-cTnT cut-off value.

All the tests were 2-tailed, and only p values < 0.05 were considered statistically significant.

Analysis was performed using SPSS (version 26.0, Statistical Package for the Social Sciences, International Business Machines, Inc., Armonk, New York, USA).

## Results

### Study population

From March 2020 to January 2021, a total of 27 patients were included (54 ± 12 (range 28–75) years and 19/27 (70.4%) were male); most significant clinical and laboratory parameters are displayed in Tables 1, 2. Most common symptoms were fever 24/27 (88.9%), cough 13/27 (48.2%) and dyspnea 6/27 (22.2%).

**Table 1:** Patients and controls characteristics

Clinical characteristic	COVID-19 (n = 27)	Control (n = 27)	p
Age, mean, years (SD)	54 (12)	58 (12)	0.263
Gender male, No. (%)	19 (70.4)	23 (85.2)	0.129
CVD comorbidities, No. (%)			
Hypertension	8 (29.6)	9 (33.3)	0.770
CAD	0	0	1
Diabetes type II	2 (7.4)	3 (11.1)	0.639
Dyslipidemia	2 (7.4)	2 (7.4)	1
Smoking	3 (11.1)	2 (7.4)	0.639

*CAD* coronary artery disease, *COVID-19* Coronavirus disease 19, *CVD* cardiovascular disease, *SD* standard deviation

**Table 2:** Patients laboratory and clinical findings

Laboratory findings, median (IQR)	Value
hs-cTnT, ng/ml	0.029 (0.007–0.057)
CRP, mg/dl	1.4 (0.43–3.93)
D-dimer, ng/dl	750 (580–1824)
WBC × 10^9^/l	5.6 (4.95–8.89)
Lymphocytes × 10^9^/l	1.06 (0.89–1.84)
Clinical findings, mean (SD)	
Respiratory rate, breaths/min	19.5 (2.2)
O_2_ sat %	97.2 (1.5)
PaO_2_/FiO_2_ ratio	383.9 (67.5)
Chest CT Score	9.6 (4.8)
Time from COVID-19 diagnosis to CMR, median (IQR), days	20.0 (13.5–31.5)

*CMR* cardiovascular magnetic resonance, *CRP* c-reactive protein, *CT* computed tomography, *hs-cTnT* High-sensitivity troponin T, *IQR* interquartile range, *O*_*2*_* sat* oxygen saturation, *PaO*_*2*_*/FiO*_*2*_ arterial oxygen partial pressure/fraction of inspired oxygen, *SD* standard deviation, *WBC* white blood cells

Increased hs-cTnT (> 0.014 ng/ml) was reported in 18/27 patients (66.6%) with a median value [interquartile range (IQR)] of 0.029 (0.007–0.057) ng/ml.

Depending on timing of symptoms’ onset^12^, all patients were classified to have a “late phase” disease (> 7 days); in addition, the median time between SARS-CoV-2 infection diagnosis and CMR was 20 (13.5–31.5) days.

Increased D-dimer (> 500 ng/ml) was found in 21/27 (77.8%) patients with a median value (IQR) of 750 (580–1823) ng/ml and increased CRP (> 0.5 mg/dl) was present in 19/27 (70.4%) patients with a median value (IQR) of 1.4 (0.43–3.93) mg/dl. WBC count was elevated (> 11.3 × 10^9^/l) in 7/27 (25.9%) patients.

Decreased lymphocyte count (< 1 × 10^9^/l) was observed in 11/27 (40.7%) patients, decreased O^2^ saturation (≤ 95%) in 5/27 (18.5%), and decreased PaO^2^/FiO^2^ ratio (< 300) in 4/27 (14.8%) patients.

Based on the Chinese CDC clinical scoring for COVID-19 [12], 1/27 (3.7%) patient were classified as severe and 26/27 (96.3%) patients as mild.

Signs of interstitial pneumonia were detected at chest CT in the vast majority of cases (25/27; 92.6%), corresponding to a mean global CT score of 9.6 ± 4.8 (range 0–15). Mean time interval between chest CT scan and CMR was 7.3 ± 3.8 days.

Underlying CVD comorbidities were present in 12/27 patients (37%), including hypertension (8/27; 29.6%) and type II diabetes (2/27; 7.4%), dyslipidemia (2/27; 7.4%). No patients had known previous CVD or history of obstructive CAD.

### CMR data

No procedural complications were observed in any patient or control, even though four patients interrupted the exam, renouncing the post-contrast MOLLI images acquisition.

CMR features are displayed in Table 3.

**Table 3:** Cardiovascular Magnetic Resonance (CMR) findings in COVID-19 patients and controls

CMR feature	COVID (n = 27)	Control (n = 27)	p
LVEDV/BSA, mean (SD), ml/m^2^	74.2 (17.4)	67.7 (11.6)	0.114
LVESV/BSA, mean (SD), ml/m^2^	37.8 (12.6)	26.1 (6.1)	< 0.001
LVSV/BSA, mean (SD), ml/m^2^	36.4 (7.1)	41.6 (8.3)	0.016
LVEF, mean (SD), %	50.3 (7.2)	61.3 (6.3)	< 0.001
LVEF < 50%, No. (%)	13 (48.2)	0 (0)	< 0.001
LV mass/BSA, mean (SD), g/m^2^	62.8 (10.9)	52.4 (9.2)	0.001
RVEDV/BSA, mean (SD), ml/m^2^	77.0 (14.2)	73.3 (14.7)	0.322
RVESV/BSA, mean (SD), ml/m^2^	40.3 (12.0)	33.2 (10.1)	0.023
RVSV/BSA, mean (SD), ml/m^2^	37.0 (7.0)	40.6 (8.6)	0.096
RVEF, mean (SD), %	48.8 (8.2)	55.7 (8.1)	0.003
Native T1, mean (SD), ms	1032 (40)	996 (29)	< 0.001
Native T1 > 1027 ms, No. (%)	11 (40.7)	3 (11.1)	0.013
T2, mean (SD), ms	52.2 (3.8)	47.2 (2.0)	< 0.001
T2 > 49.9 ms, No. (%)	14 (51.9)	0 (0)	< 0.001
ECV, mean (SD), %	28.435 (9.9)	24.3 (2.3)	0.001
ECV > 29.5%, No. (%)	10 (43.5)	1 (3.7)	0.002
Edema on STIR, No. (%)	10 (37.0)	0 (0)	< 0.001
LGE, No. (%)	12 (44.4)	0 (0)	< 0.001
Ischemic pattern, No. (%)	3 (25)	–	–
Non-ischemic pattern, No. (%)	9 (75)	–	–
Pericardial enhancement, No. (%)	2 (7.41)	0 (0)	0.15

*BSA* body surface area, *ECV* extracellular volume, *LGE* late gadolinium enhancement, *LV* left ventricle, *LVEDV* left ventricular end-diastolic volume, *LVEF* left ventricular ejection fraction, *LVESV* left ventricular end systolic volume, *LVSV* left ventricular stroke volume, *RV* right ventricle, *RVEDV* right ventricular end diastolic volume, *RVEF* right ventricular ejection fraction, *RVESV* right ventricular end systolic volume, *RVSV* right ventricular stroke volume, *STIR* short tau inversion recovery, *SD* standard deviation

LV dilation (abnormal increase of end-diastolic volume) was found in 5/27 (18.5%) patients, whereas 13/27 (48.15%) patients showed impaired LV systolic function (LVEF < 50%) and 13/27 had regional wall motion abnormalities consisting in hypo- or akinesia (the number of LV segments involved was 1–3 in four patients and 4–7 in nine patients), with a predominant involvement of mid anteroseptal and mid anterior wall segment.

A total of 20/27 (74.1) patients showed myocardial tissue signal abnormalities, including at least one of the following: increased myocardial native T1 (11/27), T2 (14/27) and ECV (10/23), areas of LGE (12/27) or edema on STIR images (10/27), or pericardial enhancement (2/27).

Nine patients had a non-ischemic-type LGE pattern, while 3 showed transmural LGE. Among cases with non-ischemic LGE pattern, eight patients showed a subepicardial and midwall distribution, whereas midwall LGE area was present in one patient; the LV wall regions involved were septum in 4/9 patients, anterior wall in 1/9, anterolateral wall in 3/9 patients, inferolateral wall in 6/9 and inferior wall in 4/9 patients.

Isolated elevation of one myocardial parametric value was found in 7/27 patients (3/27 patients with increased native T1 and 4/27 with increased T2).

**Fig. 1:**
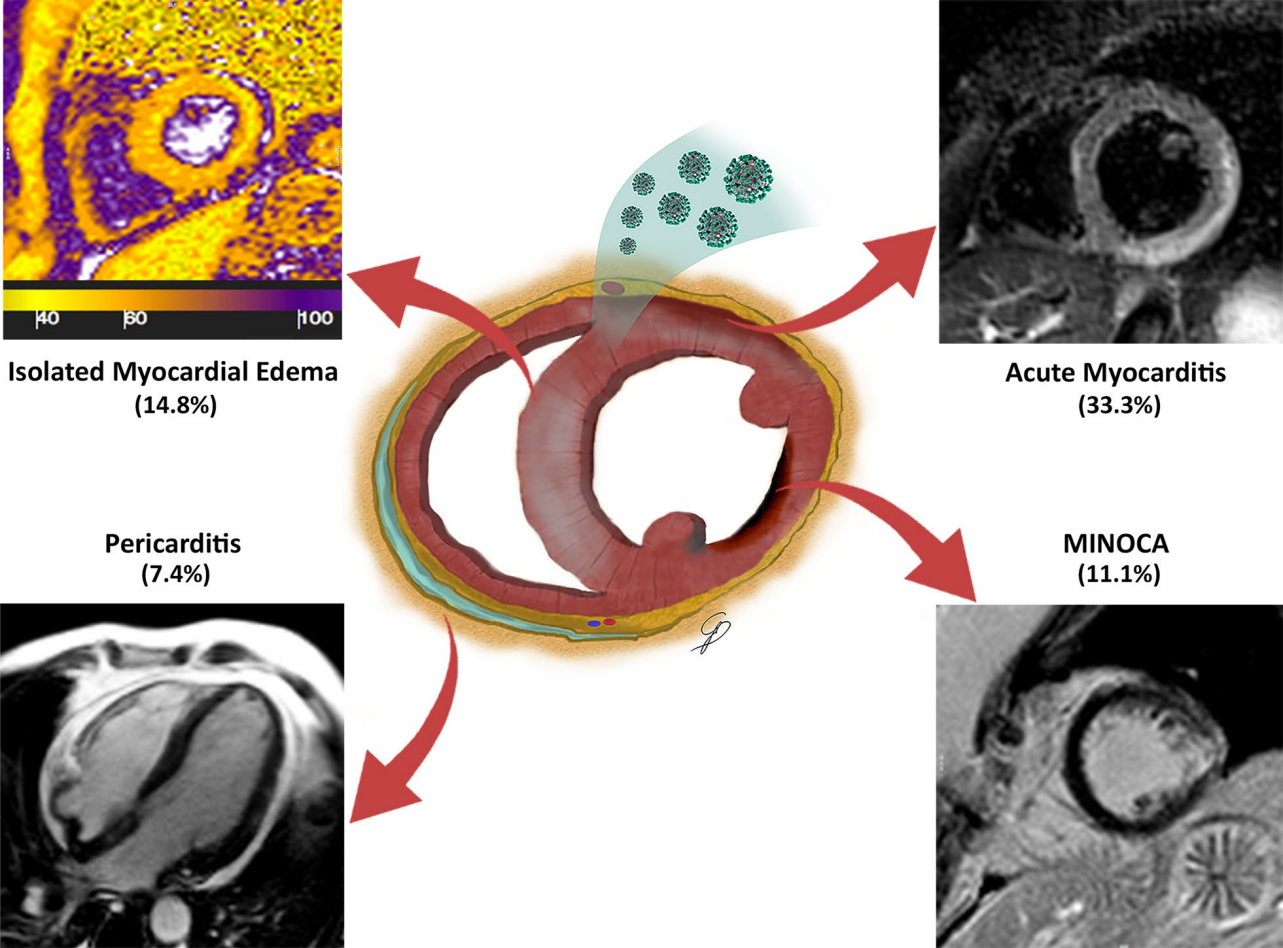
The wide spectrum of cardiac involvement in Coronavirus disease 2019 (COVID-19) patients. The drawing shows the different patterns of cardiac injury, corresponding to isolated myocardial edematous changes (upper left box, T2 map with increased T2 values), myocarditis (upper right box, short tau inversion recovery (STIR) T2 weighted image with subepicardial band of edema), pericarditis (lower left box, pericardial effusion on cine cardiovascular magnetic resonance (cMR) image) and myocardial infarction (lower right box, subendocardial enhancement on late gadolinium enhancement (LGE) image**)**

Final diagnosis of myocarditis was established in 9/27 (33.3%), pericarditis in 2/27 (7.4%) and myocardial infarction with non-obstructive coronary arteries (MINOCA) in 3/27 (11.1%) patients (Fig. 1).

Moreover, impaired biventricular contractile performance, without myocardial signal alteration, was found in 2/27 patients.

### Correlations between clinical and CMR features

A significant dependence between T2 and the hs-cTnT values was found using the Pearson’s X^2^ test (p 0.001), when these variables were considered as categorical. The correlation analysis using the Pearson Correlation index showed a moderate positive linear relationship between the T2 and the hs-cTnT values (r 0.58; p 0.002).

The linear regression analysis was conducted to examine the relationship between the T2 and hs-cTnT values. In particular the best fitting model was obtained after adding 4 parameters, hs-cTnT, WBC count, lymphocytes count, CRP value, with a r^2^ 0.59 and a r 0.77.

No significant correlation was found between the native T1 and the hs-cTnT, both considering as categorical or quantitative variables (p 0.78–0.13).

All patients with an increased ECV had an elevated hs-cTnT; with a significant dependence between the altered ECV and hs-cTnT using the Pearson X^2^ test (p 0.029) and a high degree positive correlation between these two parameters, using the Pearson Correlation Index (r 0.77; p < 0.001).

**Fig. 2:**
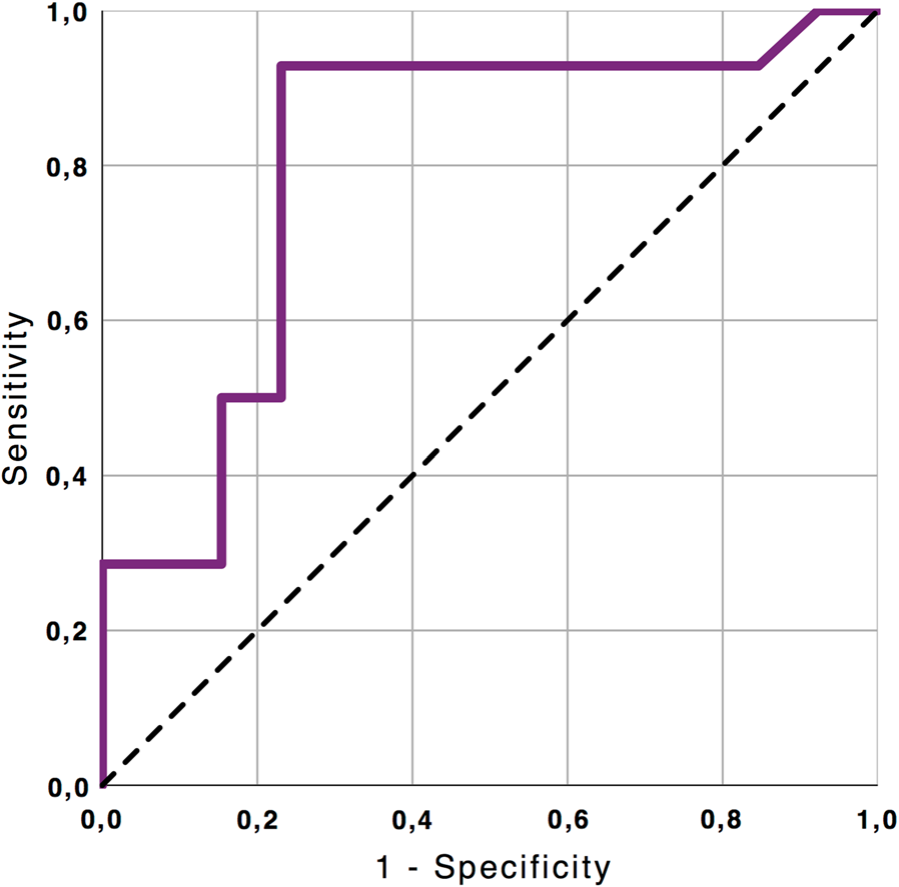
Diagnostic Performance of high sensitive troponin T (hs-cTnT) in the detection of COVID-19 related myocardial injury assessed by T2 imaging. ROC curve illustrates the diagnostic performance of hs-cTnT to detect increase of myocardial T2 value (T2 > 49.9 ms): area under curve = 0.805; 95% confidence interval: 0.626–0.984, p = 0.007). ROC, receiver operating characteristics; Hs-cTnT, high-sensitive troponin T

ROC analysis identified a hs-cTnT value > 0.0215 ng/ml as the best threshold for distinction between the healthy controls and patients with myocardial edema (sensitivity 92.9%, specificity 76.9%, p 0.007, area under the curve 0.805, Fig. 2). Adapted to all patients, this threshold identified 13 of 14 patients as having myocardial involvement on T2 maps.

**Fig. 3:**
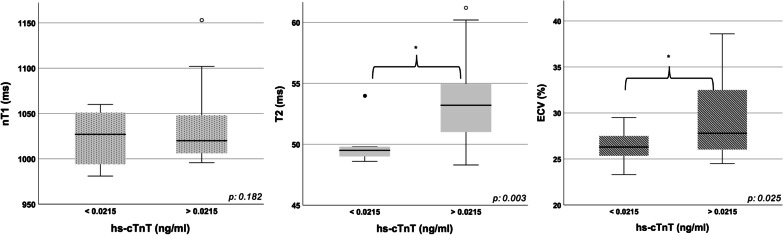
Box plot graphs of native T1, T2 and ECV comparing patients with hs-cTnT values higher or lower than 0.0215 ng/ml. Comparison between patients with high versus low hs-cTnT values in terms of native T1, T2 and ECV. In all the box plots the top of the box represents the third quartile and the bottom the first quartile. The horizontal line represents the median for entire cohort. The whiskers go from each quartile to the minimum or maximum. *p < 0.05

Applying the threshold of 0.0215, the cohort was divided into 2 groups; significant differences were found between the two groups in terms of T2 (p 0.003) and ECV (p 0.025). No significant differences were found in native T1 (p 0.182, Fig. 3).

No differences were found in CT scores between patients with abnormal values of native T1 (p 0.524), T2 (p 0.428) and ECV (p 0.807).

## Discussion

To the best of our knowledge, this is the first study using CMR to explore the nature of myocardial damage in a selected cohort of individuals with active phase of COVID-19 disease.

Besides the definition of myocardial injury, corresponding to the rise of cTnT levels above the 99th percentile upper reference limit [3], our research provides a broader insight into the possible underlying pathological substrates associated with the SARS-CoV-2 infection.

The heterogeneity of CMR patterns observed in our population reflects the complexity of disease pathogenesis, offering insight into the predominant mechanisms involved (e.g., inflammatory, cytokine-mediated, direct cytotoxicity of the virus, intra-coronary thrombosis), with potential implications on patient management and prognosis [23].

As expected, the most common CMR disease pattern we observed (33.3% of patients) was consistent with a diagnosis of clinically suspected acute myocarditis, characterized by the combination of myocardial edema with evidence of non-ischemic myocardial injury, as suggested by the new CMR diagnostic criteria [22].

CMR pattern of diffuse inflammation was more commonly detected, probably reflecting the increased interstitial macrophage recruitment and, in a limited percentage of cases, the multifocal lymphocytic infiltration, reported in previous pathological studies [24].

Coherently with the acute phase of COVID-19-related myocardial involvement characterizing our study population, the most common CMR feature observed was the increase of myocardial T2 (14/27 patients), mirroring prior studies in non-COVID-19 literature and reflecting the predominant edematous expression of the process [25].

Our average T2 values (52.2 ms) significantly differed from an earlier publication by Esposito et al. [26], reporting remarkably increased levels of T2 values (62 ms, using a reference normal value < 50 ms) in patients consecutively referred for suspected COVID-19 myocarditis. The most likely explanation of this discrepancy is the large heterogeneity of the clinical presentations observed in our cohort, which included subjects without CMR evidence of myocardial injury. An additional explanation could be the longer time interval from COVID-19 diagnosis to CMR examination in our cohort compared to their (median time of 20 days vs 3 days).

Similar observations were published by Puntmann et al. [27], who found increased T2 in 60% of 100 patients with convalescent disease stage (average interval between the last positive PCR and CMR examination was 71 days; mean T2 value 38.2 ms). Their findings suggest persistency of the inflammatory cascade beyond the acute phase of the infection, potentially triggered by the activation of an autoimmune process.

Isolated increase of native T1 values (3/27 patients), as a non-specific expression of interstitial fibrosis of unknown origin and potentially pre-existing to the SARS-CoV-2 infection, was considered non-necessarily attributable to COVID-19 related myocardial injury.

Among our COVID-19 subjects, the main differential diagnoses of acute myocarditis was pericarditis and myocardial infarction with non-obstructive coronary arteries (MINOCA). Pericardial inflammation in COVID-19 has been described as a purely inflammatory response to the systemic insult rather than a local infectious process [28]. Accordingly, the two cases of acute pericarditis in our population showed typical hallmarks of transudative pericardial effusions with associated edematous thickening of the layers [29]. MINOCA, defined by the presence of ischemic LGE with tissue edema in CMR images, not associated with epicardial coronary obstruction, was observed in three cases.

Possible explanations have been attributed to the presence of high levels of angiotensin converting enzyme 2 receptors in pericytes and endothelial cells, determining severe microvascular dysfunction enhanced by the cytokines storm [30]. Mismatch between oxygen supply and demand was reported to be a second potential mechanism of injury [31].

A further distinguished pathological CMR pattern (observed in 4 cases) showed an isolated increase of T2 without associated CMR features of myocardial damage on LGE, native T1 and ECV. This CMR phenotype, not corresponding to a diagnosis of acute myocarditis, likely represents the imaging correlate of the diffuse edematous myocardial involvement induced by the uncontrolled cytokine release, characterizing the late-phases of the infection. Presence of myocardial interstitial macrophage infiltration without myocyte injury was reported in 86% of cases from a recent multicenter study including the autopsies of 21 consecutive COVID-19 patients [24]. The authors’ hypothesis was that the characteristics of tissue damage suggest etiologies other than viral myocarditis, more likely depending on a combination of elevated proinflammatory cytokines, hypoxemia, right ventricular overload, and thrombotic complications [24].

Similar findings were published by Xu et al. [32] reporting the presence of few interstitial mononuclear inflammatory infiltrates, with no evidence of substantial damage in the heart tissue of a post-mortem biopsy.

Systemic Capillary Leak Syndrome has been advocated as a possible mechanism of pathogenesis in COVID-19 patients [33]. It causes acute loosening of the endothelial junctions, resulting in extravasation and shift of fluids, electrolytes and proteins towards the extravascular space, leading to myocardial edema [34]. This paroxysmal permeability phenomenon is frequently associated with contractile dysfunction and tends to regress generally without any permanent sequelae. This might explain the increase of ECV found in our population, which correlated with hs-cTnT. As a further confirmation of this hypothesis, we found a positive linear correlation between ECV and T2 (r^2^: 0.75; p < 0.001).

CMR follow-up data are missing in literature with these regards and will certainly provide better comprehension of the underlying mechanism of injury and its transient nature with or without permanent tissue abnormalities.

Interestingly, two patients showed newly diagnosed mild reduction of biventricular systolic function in absence of myocardial signal alterations both in conventional sequences (T2 STIR and LGE) and in relaxometric imaging. Interpretation of this finding remains questionable as a pre-existent ventricular impairment cannot be excluded and could not be differentiated from a chronic evolution of myocardial damage.

### Correlation between CMR parameters and hs-cTnT values (clinical variables)

We found a positive linear relationship between the T2 mapping and hs-cTnT values.

Besides myocardial necrosis, hs-cTnT assays can be detectable in COVID-19 as the consequence of the transient ischemic or inflammatory conditions associated with the disease, including respiratory and renal failure, hypoxemia, tachyarrhythmias and thrombo-embolic disease [35].

Regardless of the underlying pathogenetic mechanisms, troponin levels have been hypothesized to be the expression of a general hyperinflammatory status [36]. At the level of the heart, this leads to the accumulation of interstitial edema, which proportionally increases T2, explaining the positive correlation observed. The increase of T2, indeed, is able to identify 13 out of 18 patients with hs-TnT level above the 99th percentile, even as isolated CMR feature (4/18 patients), representing the best predictor of myocardial injury.

Coherently, the stepwise linear regression analysis showed that the increase of each of the inflammatory biomarkers (WBC, lymphocyte count and CRP) values, was associated with higher T2; these results confirm that patients with myocardial injury showed a more pronounced inflammatory response with direct impact on T2 measurements.

ROC analysis indicated that a hs-cTnT value > 0.0215 ng/ml would define the best cut-off value to differentiate normal versus increased T2 (sensitivity 92.9%, specificity 76.9%). This threshold identified 13/14 patients as having evidence of myocardial pathological involvement on T2 and may represent a potential cut-off value to direct patients to CMR examination.

A further interesting finding is the non-correlation between the extent of pulmonary disease and occurrence of myocardial involvement, which further supports the theory that cardiac involvement is not affected or a complication of pulmonary pathology, even though they likely share common pathogenic mechanisms.

### Limitations

Our sample size was modest, but nonetheless represents, up to date, one of the largest cohorts of patients who have undergone CMR with *active* COVID-19.

Because of the complexity of the diagnostic exam, requiring repeated breath holds and an average scanning time of 50 min, we had a selection bias regarding the clinical stage of patients enrolled, almost all presenting with a mild form of disease.

Myocardial injury proportionally correlated with the severity of the clinical manifestations of COVID-19 and potentially identifies patients with worse baseline clinical status. This has an obvious impact on the prevalence and extent of tissue damage observed in our study.

Similarly, no early phase disease patients (i.e., less than 7 days after positive PCR) were included in our cohort. This indirectly confirms that myocardial involvement begins or persists days after disease onset.

Our study did not provide prognostic data, which are likely to further refine the role of CMR imaging in this complex clinical scenario and to better elucidate possible inclusion criteria for patient’s selection.

None of our patients performed an endomyocardial biopsy, therefore the diagnosis of myocarditis in our patients should be considered as not definitively confirmed.

Finally, we did not include asymptomatic COVID-19 individuals, in which multiparametric imaging data would allow subclinical detection of structural damage with potentially relevant implications on early diagnosis and clinical decision-making.

## Conclusions

CMR allows recognition and characterization of myocardial damage in a cohort of selected COVID-19 individuals with active disease. This consists in heterogeneous patterns of injury ranging from acute myocarditis, to MINOCA, pericarditis, and CMR evidence of isolated edematous changes. The prompt recognition of pattern disease is pivotal to drive therapy and patient’s management. Myocardial T2 appears to be the prevalent CMR imaging biomarker in active COVID-19 patients and the best predictor of myocardial injury.

## Supplementary Information

**Additional file 1: Table S1.** CMR scanning protocol parameters in detail.

## Data Availability

All data generated or analyzed during this study will be available at the request of the referees and it will be included in the published article as supplementary information files, once the manuscript is accepted.

## Abbreviations

ACS: Acute coronary syndrome
AHA: American Heart Association
BSA: Body surface area
bSSFP: Balanced steady state free precession
CAD: Coronary artery disease
CDC: Center for Disease Control
CE: Contrast enhanced
CMR: Cardiovascular magnetic resonance
COVID 19: Coronavirus disease 2019
CRP: C-reactive protein
CT: Computed tomography
cTnT: Cardiac troponin T
CVD: Cardiovascular disease
DWI: Diffusion weighted imaging
ECV: Extracellular volume fraction
EDV/BSA: End-diastolic volume/body surface area
EF: Ejection fraction
FLASH 3D: Fast low angle shot 3D
hs-cTnT: High-sensitive troponin T
IR-CE T1w: Contrast-enhanced inversion recovery T1-weighted
LGE: Late gadolinium enhancement
LV: Left ventricle/left ventricular
LVEDV: Left ventricular end-diastolic volume
LVEF: Left ventricular ejection fraction
LVESV: Left ventricular end-systolic volume
LVSV: Left ventricular stroke volume
MI: Myocardial infarction
MINOCA: Myocardial infarction with non-obstructive coronary arteries
MOLLI: Modified Look-Locker inversion-recovery
PaO_2_/FiO_2_: Arterial oxygen partial pressure/fraction of inspired oxygen
PPE: Personal protective equipment
ROC: Receiver operating characteristic
RT-PCR: Reverse transcription–polymerase chain reaction
RV: Right ventricle/right ventricular
RVEDV: Right ventricular end-diastolic volume
RVEF: Right ventricular ejection fraction
RVESV: Right ventricular end-systolic volume
RVSV: Right ventricular stroke volume
SARS-CoV-2: Severe acute respiratory syndrome Coronavirus 2
SD: Standard deviation
SI: Signal intensity
STIR: Short tau inversion-recovery
T1w: T1 weighted
WBC: White blood cells
X^2^: Chi-squared
