# Supplementary material for: T2-mapping increase is the prevalent imaging biomarker of myocardial involvement in active COVID-19: a Cardiovascular Magnetic Resonance study
Source: J Cardiovasc Magn Reson. 2021 Jun 10;23:68. doi: 10.1186/s12968-021-00764-x (PMC8189727; doi:10.1186/s12968-021-00764-x)
Supplement: Supplementary file 1 — Additional file 1: Table S1. CMR scanning protocol parameters in detail. [file 12968_2021_764_MOESM1_ESM.docx]

| **Sequence** | **TR (msec)** | **TE (msec)** | **FA (°)** | **TI (msec)** | **Slice Thickness (mm)** | **FOV (mm^2^)** | **Matrix** |
| --- | --- | --- | --- | --- | --- | --- | --- |
| **STIR** | 2 R-R intervals | 75 | 180 | 170 | 8 | 340x400 | 256x256 |
| **MOLLI (T1 mapping)** | 314 | 1.12 | 35 | 200 | 8 | 340x400 | 256x256 |
| **T2-prep (T2 mapping)** | 239 | 1.13 | 12 | n/a | 8 | 340x400 | 256x256 |
| **Cine-bSSFP** | 51.3 | 1.21 | 45 | n/a | 8 | 340x400 | 256x256 |
| **IR (LGE)** | 9.6 | 4.4 | 35 | 250-300 | 8 | 340x400 | 256x256 |

**CMR scanning protocol parameters in detail** – *TE: echo time; FA: flip angle; FOV: field of view; IR: inversion recovery; TI: inversion time; LGE: late gadolinium enhancement; MOLLI: modified look locker; TR: repetition time; bSSFP: balanced steady state free precession; STIR: short tau inversion recovery.*
